# Analysis for Distinctive Activation Patterns of Pain and Itchy in the Human Brain Cortex Measured Using Near Infrared Spectroscopy (NIRS)

**DOI:** 10.1371/journal.pone.0075360

**Published:** 2013-10-03

**Authors:** Chih-Hung Lee, Takashi Sugiyama, Aiko Kataoka, Ayako Kudo, Fukue Fujino, Yu-Wen Chen, Yuki Mitsuyama, Shinobu Nomura, Tohru Yoshioka

**Affiliations:** 1 Department of Dermatology, Kaohsiung Chang Gung Memorial Hospital and Chang Gung University College of Medicine, Kaohsiung, Taiwan; 2 Advanced Research Institute for Bioscience, Waseda University, Tokyo, Japan; 3 Department of Dental Hygiene, Kanagawa Dental University Junior College, Yokosuka, Japan; 4 Department of Nuclear Medicine, Kaohsiung Medical University, Chung-Ho Memorial Hospital, Kaohsiung, Taiwan; 5 Graduate School of Human Sciences, Waseda University, Saitama, Japan; 6 Faculty of Human Sciences, Waseda University, Saitama, Japan; 7 Graduate Institute of Medicine, Kaohsiung Medical University, Kaohsiung, Taiwan; Tokai University, Japan

## Abstract

Pain and itch are closely related sensations, yet qualitatively quite distinct. Despite recent advances in brain imaging techniques, identifying the differences between pain and itch signals in the brain cortex is difficult due to continuous temporal and spatial changes in the signals. The high spatial resolution of positron emission tomography (PET) and functional magnetic resonance imaging (fMRI) has substantially advanced research of pain and itch, but these are uncomfortable because of expensiveness, importability and the limited operation in the shielded room. Here, we used near infrared spectroscopy (NIRS), which has more conventional usability. NIRS can be used to visualize dynamic changes in oxygenated hemoglobin and deoxyhemoglobin concentrations in the capillary networks near activated neural circuits in real-time as well as fMRI. We observed distinct activation patterns in the frontal cortex for acute pain and histamine-induced itch. The prefrontal cortex exhibited a pain-related and itch-related activation pattern of blood flow in each subject. Although it looked as though that activation pattern for pain and itching was different in each subject, further cross correlation analysis of NIRS signals between each channels showed an overall agreement with regard to prefrontal area involvement. As a result, pain-related and itch-related blood flow responses (delayed responses in prefrontal area) were found to be clearly different between pain (*τ* = +18.7 sec) and itch (*τ* = +0.63 sec) stimulation. This is the first pilot study to demonstrate the temporal and spatial separation of a pain-induced blood flow and an itch-induced blood flow in human cortex during information processing.

## Introduction

Despite great strides in understanding the neuronal processing of pain over the last several years, the underlying mechanism of dynamic processing in the brain cortex remains a fundamental question [1]. Compelling evidence accumulated over the past decade indicates that multiple cortical and subcortical areas are coordinated in pain perception in humans [2]. The brain circuitry for processing acute pain may involve the same cortical areas involved in emotional learning and memory [3].

Distinct from pain, itch is an unpleasant perception that evokes the desire to scratch. Although there are dissimilarities between their processing, pain and itch are thought to be closely related in that weak activation of nociceptors mediates itch, while strong activation of the same receptors results in weak pain [2]. Moreover, there is a broad overlap in neuromediators of pain and itch signal processing. Interestingly, scratch-induced pain can abolish itching [4], suggesting reciprocal control of pain and itch.

In the 1990’s, functional human brain imaging utilizing positron emission tomography (PET) and functional magnetic resonance imaging (fMRI) was developed based on measurements of the dynamic changes in cerebral blood flow. These studies demonstrated that neural processing of nociception and pain signals occur in several brain regions operating in parallel [5], [6], [7], [8], [9]. Vierow et al. studied the effect of itching on cortical and subcortical brain regions, but found no significant difference between the itch and control conditions using fMRI based on blood-oxygenation level dependent (BOLD) signals [10]. These imaging studies provided excellent information, but MRI apparatus is expensive and non-portable, and the operation is limited in the magnetically-shielded room.

Near infrared spectroscopic (NIRS) topographic imaging, based on the measurement of oxygenated hemoglobin (HbO2) and deoxygenated hemoglobin (HbR) concentrations, was recently developed to obtain dynamic brain images that overcome the limited operation in the shielded room of PET and fMRI [11]. Initial NIRS studies relevant to pain were performed independently by Kell et al. and Durkin et al. by measuring oxygenated blood flow in muscle [12], [13]. The fact that neuronal network activation is always accompanied by an increase in regional blood flow is the principle underlying NIRS brain imaging as well as fMRI.

In the present study, we applied NIRS imaging to compare the dynamic activation pattern of cortical areas between pain- and itchy-related information processing. For pain stimulation, NIRS images suggested on first appraisal that the activated area was changing rapidly in the frontal cortex and the activation pattern was largely different among each subjects. However, on closer inspection, there was a statistical difference found in time-dependent cross correlation of NIRS signals between the central region and peripheral region of the prefrontal cortex (p<0.05 by cross correlation method). Although itch-activated NIRS signals were well correlated between those regions, there was no or a miniscule time-lag between them. This suggested differences in how the prefrontal cortex processed information from pain and itch signals.

## Materials and Methods

### Subjects

Seven healthy human subjects (2 men [M1–M2] and 5 women [F1–F5], age 25–38 years) were enrolled in the study upon providing written consent. Subjects with past history of atopic diathesis, including allergic rhinitis, asthma, atopic dermatitis, and allergic conjunctivitis were excluded. In addition, subjects with past history of contact hypersensitivity and urticaria were both excluded. All subjects were right-handed. Subjects with excessive Holmes and Rahe Stress Scale (>100) were excluded [14]. The subjects were told to relax and lie down for 30 minutes. Th pain and itch stimulations as well as the following NIRS measurements were performed in a quiet, temperature controlled (23–25°C) and humidity-controlled (40–50%) room in the morning (9:00–11:00 AM). All experimental procedures were approved by the Ethics Committee of Kanagawa Dental University. Each subject was seated in a reclining chair with their right arm placed on a side table. Pain stimulation of various intensities was applied to the tip of the right index finger using a push-pull digital force gauge (Aikoh Engineering Co., Osaka Japan). The intensity of the stimulation was set to 0.7 N, 1.0 N, 1.4 N, 2.1 N, and 3.0 N. For pain induction, each metered stimulus was applied for 5 seconds and then paused for 175 seconds. The cycle (180 seconds) was repeated seven times (each with a different force) in each subject. Itch stimulation was applied to the right forearm using the pin-prick test with a histamine solution (1 mM, Sigma-Aldrich, St. Louis, MO). The histamine was delivered into the forearm skin via the skin prick and an itch sensation was generated in the subjects. The histamine was wiped off after the itch sensation had faded. Before and after itch stimulation by a skin prick, a scratch stimulation was applied nearby using a nail for 15 seconds. During each experiment, subjects were requested to point score the pain and itch intensity on a graded visual analogue scale (VAS) at 3-minute intervals. The whole process was repeated on three different days.

### Near Infrared Spectroscopic (NIRS) Topography

**Figure 1:**
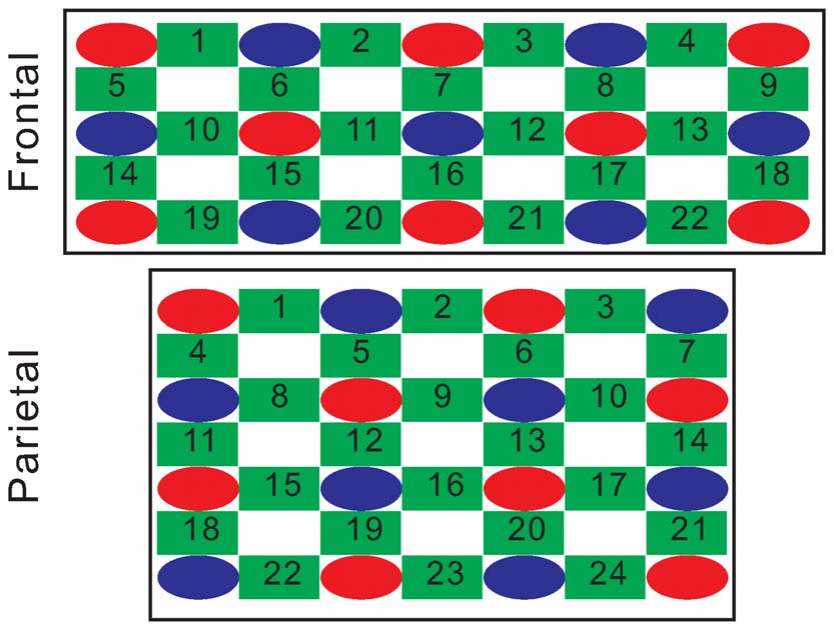
Channel network arrangement for near infrared spectroscopic (NIRS) topography. The optical topography system ETG-4000 comprised 16 elements for the NIR light source (red) and 15 elements for the detector (blue). The numbers represented the orders of the red, blue, and green elements. Each emitter and detector was inserted into a standard holder in a grid-like arrangement. The emitters and detectors were arranged in a 3×5 matrix for the frontal area and a 4×4 matrix for the parietal area, and the whole array was set on the frontal and parietal scalp of the subject. The change in [HbO2] and [HbR] at each channel (green; 22 for the frontal area and 24 for the parietal area) was acquired and calculated using this arrangement. The signal in the blank area (white) was measured and calculated from the four surrounding channels.

Brain imaging was performed using an optical topography system ETG-4000 (Hitachi Medical Co., Tokyo) with 22 channels for the frontal area and 24 channels for the parietal area. The operator checked if the NIR sensors were closely applied to the scalp of the subjects using an established standardized process according to the manufacturer’s instructions. The NIRS system generates NIR at 780 and 830 nm, which is transmitted via optical fiber bundles to the brain through the cranium via an emitter (Fig. 1; red ovals). The scattered and reflected light was sampled by NIR sensors (Fig. 1; blue ovals) placed on the scalp 30 mm away from the NIR emitters. The NIR emitters and sensors were arranged on the head as shown in Figure 1. The signal between each emitter and receiver, called a channel (green squares), was measured at a given time point and reflected changes in the concentrations of oxyhemoglobin ([HbO2]) and deoxyhemoglobin ([HbR]), differentiated by the distinctive absorption spectra of HbO2 and HbR, The signal in the blank area (white) was measured and calculated from the four surrounding channels. Total hemoglobin concentration ([HbT]) was calculated by adding [HbO2] and [HbR]. All experiments were initiated after confirming the baseline [HbO2] and [HbR] levels. The relative dynamic changes in [HbO2] data were recorded for 22 channels in five lines in the front and 24 channels in 7 lines in the rear simultaneously, generating two-dimensional optical topographic images of changes in [HbO2].

### Data Analysis

Quantitative optical data were collected using a personal computer. Baseline [HbO2] values were calculated by averaging the data for 30 seconds before the start of the stimulus. Signal comparison was performed by calculating the cross correlation function between channels. The cross correlation is a measure of temporal, not spatial, similarity of two waveforms as a function of a time-lag applied to one of them and has applied to neurophysiology [15], [16], [17], [18], [19].

[HbO2] NIRS trains from two channels are represented as the time series *X*(*t*) and *Y*(*t*). The cross-correlation function between the trains *X*(*t*) and *Y*(*t*) is calculated as follows:
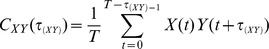



Where *T* is a number of measure points in the time series and *τ_(XY)_* is a time-lag between *X*(*t*) and *Y*(*t*). The value of *C_XY_*(*τ_(XY)_*) represents the variation of the [HbO2] NIRS signal from channel *Y*, conditioned on the fact that the [HbO2] NIRS signal from channel *X* changes *τ_(XY)_* second earlier. In the present study, to calculate the cross-correlation function of channel Y(ChY) against channel X (ChX), waveform of ChY was slid against ChX from −60 sec to +60 sec at every 100 msec and the correlation coefficient was calculated for each sliding time. A delay time that showed a maximum correlation coefficient was defined as a time lag (tau) for ChY against ChX. To remove physiological fluctuation, the raw data of [HbO2], [HbR], and [HbT] from individual channels were smoothed by moving average (average from −0.2 sec to +0.2 sec).

### Statistical Analyses

Significance was assessed using the Kruskal-Wallis test followed by the Mann-Whitney’s U test (α = 0.05). The significance level was set at *p*<0.05.

## Results

### Typical Dynamic Trace Pattern Evoked by Pain Stimulation Recorded Using NIRS Imaging

**Figure 2:**
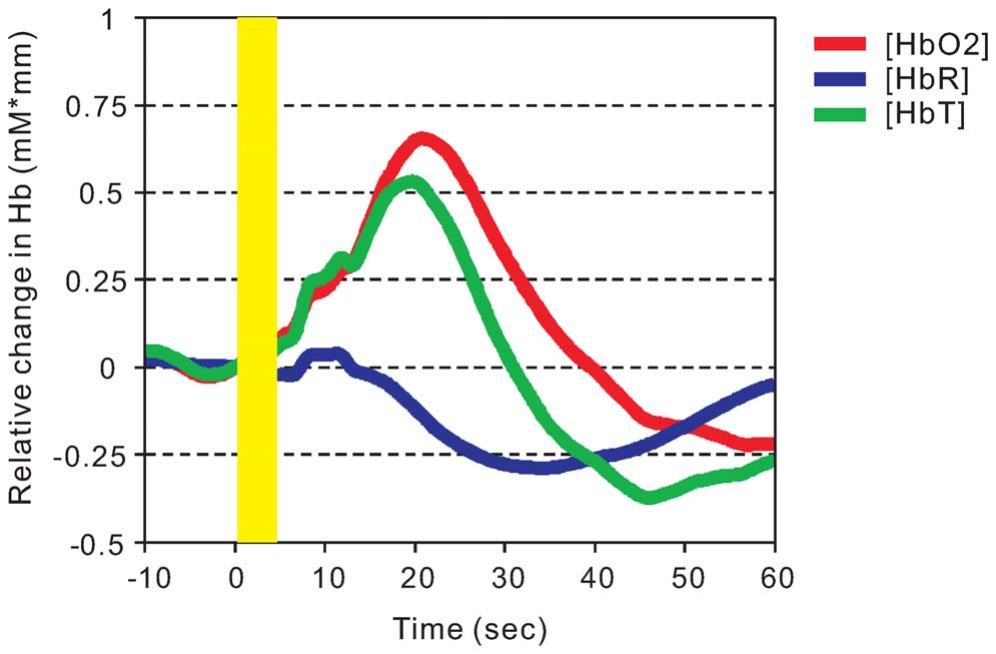
Typical dynamic responses recorded from each channel after pain stimulation. A typical response from channel 11 exhibited dynamic changes in [HbO2] (red), [HbR] (blue), and [HbT] (green). The pain stimulus (5 seconds) was applied to the tip of right index finger at the time indicated by the yellow bar.

In a typical response to pain, the [HbO2] exhibited a transient increase immediately after stimulation, a gradual increase to a peak at approximately 20 seconds after pain stimulation, and then an exponential reduction to baseline (less than resting level; Fig. 2). The [HbR], on the other hand, decreased slightly. The time to reach the lowest [HbR] (35 seconds) was longer than that required to reach the highest [HbO2] (20 seconds).

### Dynamic NIRS Responses in Frontal and Parietal Areas After Pain Stimulation

**Figure 3:**
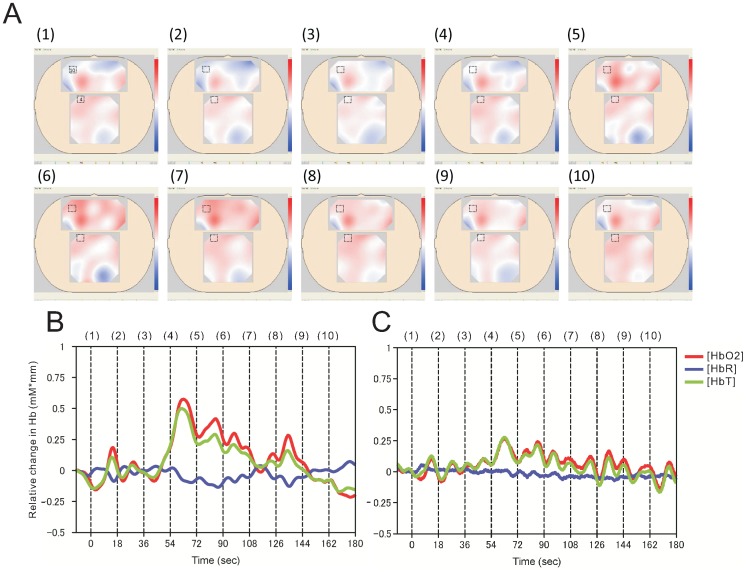
Dynamic brain cortex responses and imaging after pain stimulation. (A) Ten sequential NIRS images, obtained every 18 seconds, and numbered from 1 to 10. Red indicates that [HbO2] was above the level in the resting state and blue indicates that [HbO2] was lower than the resting level. Changes in [HbT] and [HbR] are not included in the present NIRS images. The small rectangles in the frontal and parietal areas correspond to the location of channel 10 and 4, respectively. (B) The dynamic trace recorded from channel 10 in the frontal area. The numbers 1 to 10 along the upper Y axis correspond to the numbered images in A. (C) The dynamic trace recorded from channel 4 in the parietal area. The numbers 1 to 10 along the upper Y axis correspond to the numbered images in A.

Ten serial images for subject III based on the optical data from channel 10 of the frontal area and channel 4 of the parietal area were obtained at a sampling rate of 0.06 frames/second after pain stimulation (Fig. 3A). Approximately 10 seconds or more after stimulation, a relatively small peak of [HbO2] appeared in the frontal and parietal areas (Fig. 3B,C). Approximately 60 seconds after stimulation, a 2nd peak developed in the frontal area, while the response in the parietal area remained blunted and oscillatory. The decrease in [HbR] was relatively small compared with the increase in [HbO2]. Oscillations of approximately 0.08 Hz in the [HbO2] response were observed in both the frontal and parietal areas.

### Dose-dependent Responses of [HbO2] to Pain Stimulation

**Figure 4:**
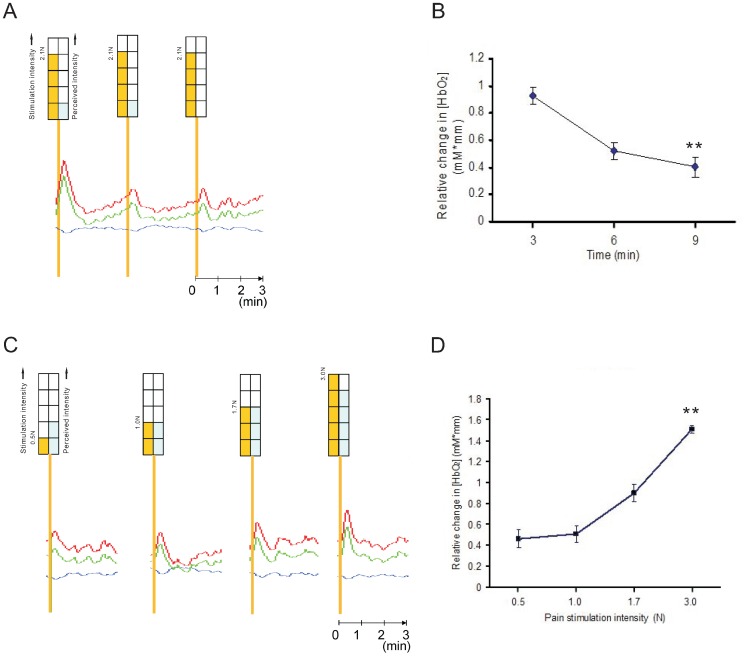
Sequential responses of channel 10 in the frontal area to constant or increasing pain intensities. (A) Responses to sequential applications of constant pain stimulation. The yellow bar represents the pain stimulation intensity (2.1 N) and the blue bar represents the subjective pain score (VAS). Red, blue, and green traces of the dynamic response represent [HbO2], [HbR], and [HbT], respectively. The traces are from channel 10 in the frontal area of subject M-1. (B) Mean (n = 4 subjects) peak responses in [HbO2] in channel 10 in the frontal area. NIRS signals were significantly decreased when constant intensities of stimulation were given (**p<0.05, vs the first response). Error bars represent SD. (C) Responses to applications of increased pain stimulation. The yellow bar represents the pain stimulation intensity (0.5 N, 1.0 N, 1.7 N, and 3.0 N) and the blue bar represents the subjective pain score (VAS). Red, blue, and green traces of the dynamic response represent [HbO2], [HbR], and [HbT], respectively. The traces are from channel 10 in the frontal area of subject M-1. (D) Mean (n = 4 subjects) peak responses in [HbO2] in channel10 in the frontal area when pain stimulation intensity was gradually increased (**p<0.05, vs the response in the first stimulation (0.5 N)). Error bars represent SD.

To determine whether subjects perceived greater pain following stronger stimulation, we examined response to a series of stimulations of constant or gradually increasing intensity for each subject. Three successive constant intensities (2.1 N) of stimulation resulted in gradually decaying reported perceived pain and NIRS response in channel 10 in the frontal area, which may be due to adaptation or habituation (Fig. 4A,B). Stimulation with gradually increasing intensity resulted in a corresponding increase in the peak [HbO2] (Fig. 4C). Thus, there was a dose-dependent response in frontal cortex to increasing pain stimulation.

### Pain Activated Signals in the Frontal and Parietal Areas

**Figure 5:**
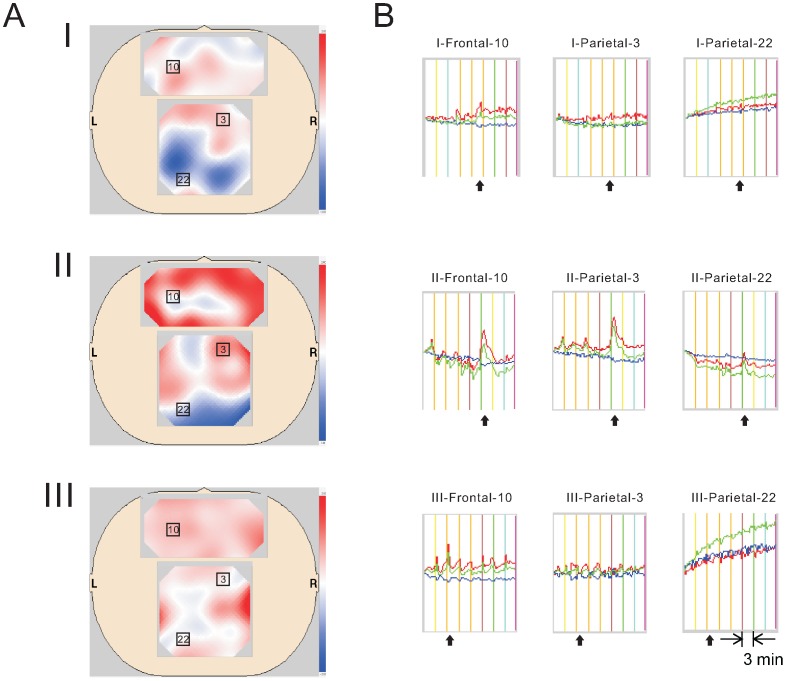
Dynamic NIR imaging after 7 random-intensity sequential pain stimulations. (A) The image collected during which the subject reported the most pain is shown for each of 3 subjects (I, II, and III). Among the three subjects according to the relationship between the stimulation and the NIRS response, subject M-1 (I) exhibited the smallest response, subject F-2 (II) exhibited a strong response, and subject M-2 (III) exhibited the clearest response in the frontal area. The rectangles indicate the location of the channels for which the dynamic pattern is shown in B: channels 10, 11, 12, and 13 in the frontal area, and channels 3 and 22 in the parietal area. (B) The dynamic pattern from channel 10 in the frontal area is shown in I-Frontal-10, II-Frontal-10, and III-Frontal-10. Channel 3 in the parietal area exhibited similar but smaller responses compared with those in the frontal area (I-Parietal-3, II-Parietal-3, and III-Parietal-3). Channel 22 recorded an unrelated pattern (I-Parietal-22, II-Parietal-22, and III-Parietal-22). Dynamic changes in other channels in the parietal area exhibited a similar response with intensities between those recorded in channel 3 and 22. The time interval between pain stimulations was 3 minutes. Arrows indicate the time points when the subjects reported the most pain, corresponded to the NIRS images shown in A. The different color bars represent pain stimulations of different intensities.

We examined the correlation of NIRS signals between frontal and parietal areas after 7 pain stimulations of different intensities. The pain-stimulation-dependent change in [HbO2] was obtained from three subjects on the same day, each given a different random order of pain stimulation intensities. Each subject showed a different response pattern in the frontal area to the set of stimulations (Fig. 5), ranging from clear to very small magnitude responses. After 3-minute pre-recording, the cycle of 5-second pain stimulation and resting period (175 sec) were repeated seven times. Each pain stimulus was applied at the time indicated by the color bars in Figure 5B. Responses in the parietal area were similar among subjects, but very small in magnitude. Based on the NIRS signal intensity, these findings suggested that the parietal and frontal areas received the same type of pain signal, but the brain responses were higher magnitude in the frontal area. This same experiment was repeated in the same subjects on another day and the results were consistently reproduced (The intra-class correlation coefficient is significantly high >0.75).

### Functional Neural Network in the Frontal Area for Pain Signal Processing

**Figure 6:**
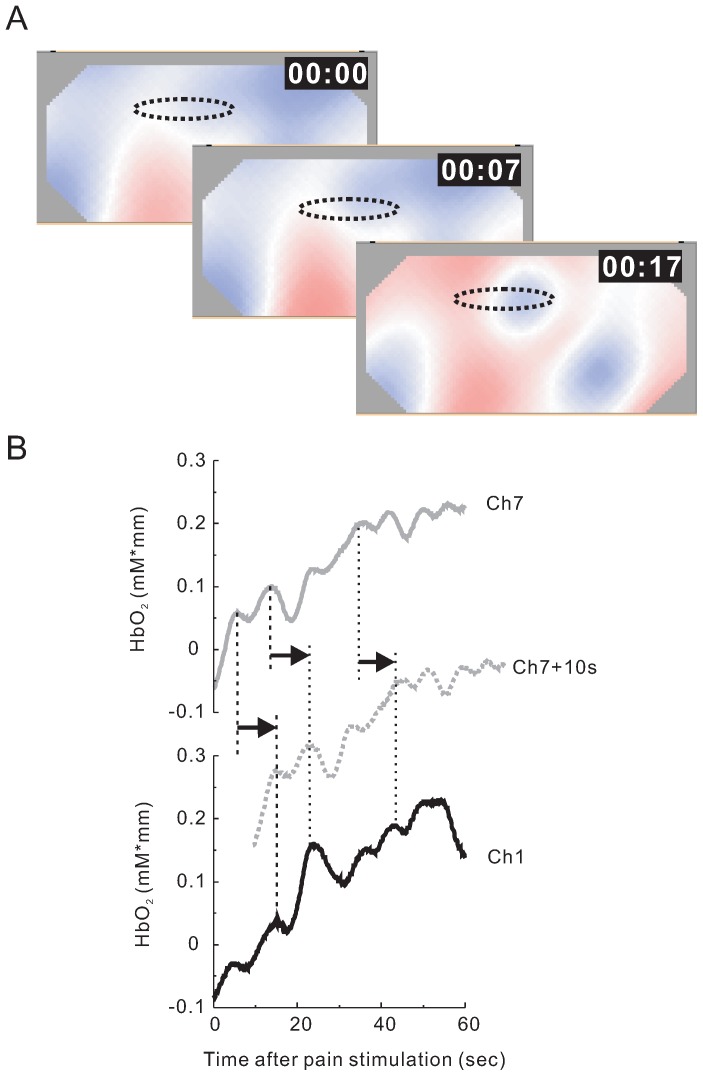
Comparison of [HbO2] NIRS data between frontal region and temporal region at prefrontal area. (A) Three sequential HbO2 NIRS images obtained 0, 7, 17 seconds after pain stimulation. A dotted circle indicates the area that [HbO2] was initially increased. Changes of [HbO2] was sequentially occurred from frontal region to temporal region (arrows indicate the direction of flow of [HbO2] change). (B) Typical time traces of NIRS data of frontal region (CH7) and temporal region (Ch1) at prefrontal area for pain stimulation. Representative data were [HbO2] traces of CH7 (gray line) and that of CH1 (black line) after pain stimulation from subject I. The sliding of the trace of CH7 toward +10 sec (broken gray line) revealed that peaks of [HbO2] in CH7 preceded those in CH1.

**Figure 7:**
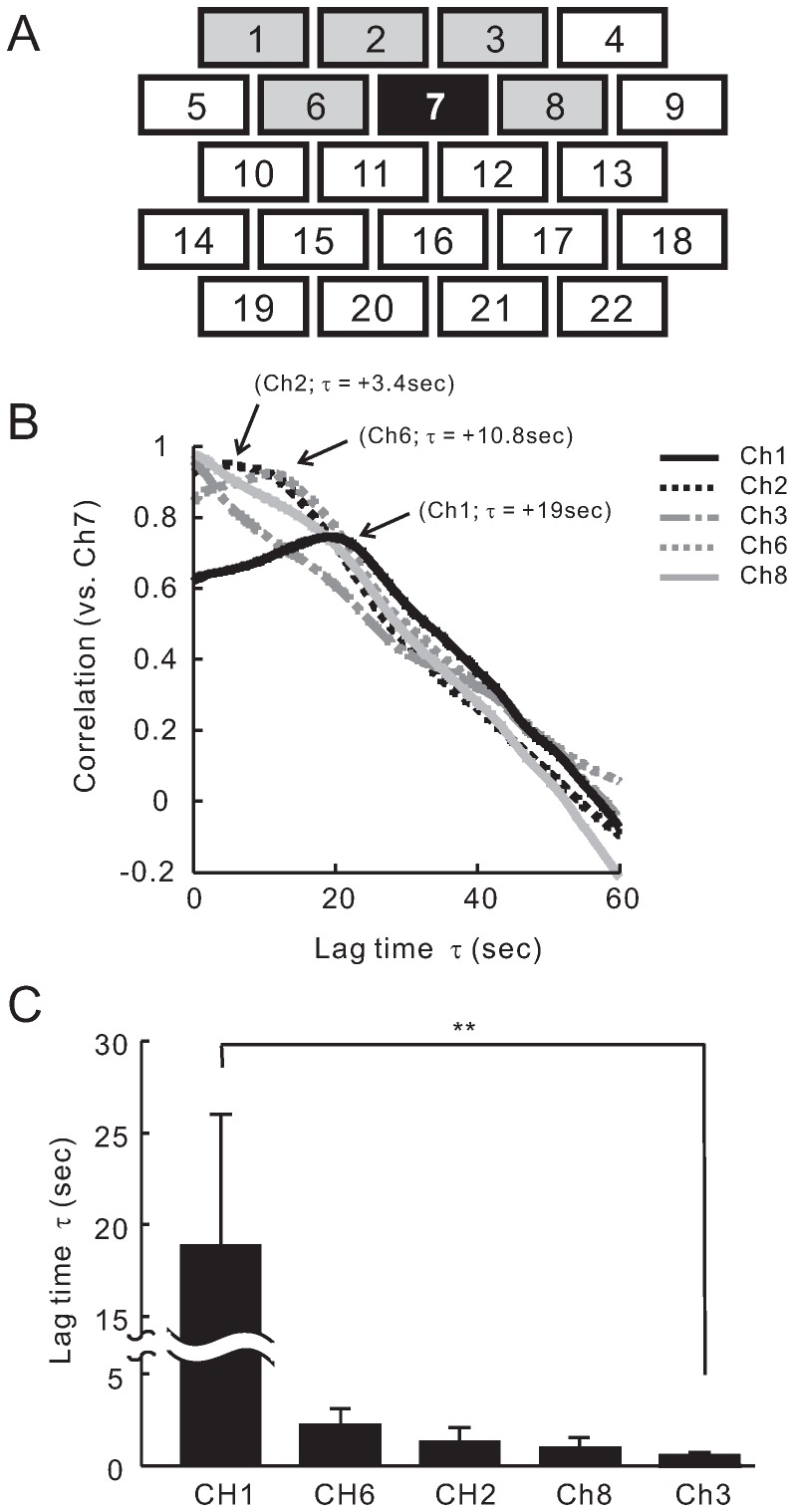
Cross correlation analysis of [HbO2] NIRS data from each channels at prefrontal area for pain stimulation. (A) Cross correlation function of [HbO2] traces from each channel against those from Ch7 for pain (left) was calculated. Average correlation coefficient was 0.62, with grey channels exhibiting a significant correlation (coefficient was over 0.7). (B) Representative data shown are from subject III. Note, the cross correlation analysis revealed that the correlation peak delay (*τ* = +19 sec for Ch1, +10.7 sec for Ch6, +3.4 sec for Ch2) was observed in pain stimulation. (C) Averages of the time-lag between well correlated channels (14 trials from 4 subjects) were calculated for pain stimulation. Data are shown as Mean ± SD. Difference in the lag time between Ch1 and Ch3 (none delay channel) was significant. ^**^ *P*<0.01.

In order to decide the most effective method analyzing moving and changing pattern with time, we observed time-dependent NIRS pattern at prefrontal cortex in pain stimulation. As shown in Figure 6A and S1, images after pain stimulation showed that the change of NIRS signals in prefrontal cortex occurred originally in the center of prefrontal area (Ch6, Ch7 and Ch8), and the signals changed later in the peripheral area (Ch1 and Ch2). We compared the traces of [HbO2] NIRS signals at Ch7 and Ch1 from subject III, and observed similarity in the dynamic pattern of [HbO2] NIRS signals between these channels as the trace of the NIRS signals from Ch7 slid positively for 10 sec (Fig. 6B). Next, we calculated the time-dependent cross correlation function between Ch7 and other channels for 4 subjects. Our results showed channels 1, 2, 3, 6 and 8 to have a significant correlation with Ch7 (correlation coefficient was over 0.7; average of correlation coefficient was 0.62; Fig. 7A). An example of cross correlation function for well correlated channels from subject III is shown in Figure 7B. Remarkably, the correlation peak delays were different among these channels. Delay time of Ch1 was large (*τ* = +19 sec) and that of Ch2 and Ch6 were middle (*τ* = +10.7 sec and +3.4 sec, respectively). No delay was observed at both Ch3 and Ch8. Averages of delay time for each channel from the four subjects are summarized in Figure 7C. It is important to note that [HbO2] NIRS signals of Ch1 exhibits a similar waveform to those of Ch7 with large delay (*τ* = +19.2 sec). These results suggest a direction of pain signal information flowing from center of prefrontal area (Ch7) to peripheral area (Ch1). Therefore, by correlating responses from channels in prefrontal cortex, we could identify possible time-dependent connections between regions.

### Dynamic Change in [HbO2] and [HbR] Induced by Itch Stimulation

**Figure 8:**
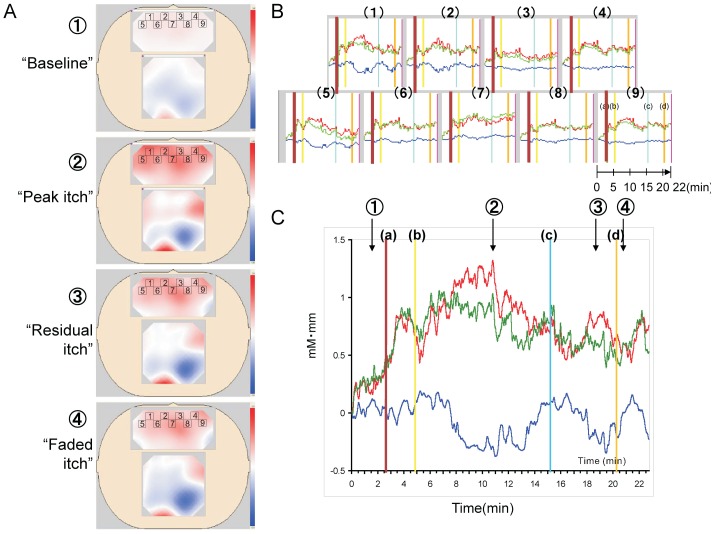
Brain imaging and dynamic signals in response to long-lasting itch stimulation. (A) Typical NIRS images of (1) pre-stimulation, (2) peak itch sensation, (3) residual itch, and (4) faded itch sensation. The small rectangles in the frontal areas correspond to the location of channel 1 through 9. (B) The traces of channels 1 through 9 in the frontal area showed dynamic changes in [HbO2] (red), [HbR] (blue), and [HbT] (green) with itch stimulation. (C) An expanded view of channel 1. (a) time of 1st scratch stimulus, (b) time of skin prick and histamine application, (c) time of wiping off histamine solution, and (d) time of 2nd scratch stimulus. Times indicated by 1,2,3, and 4 corresponded to the images shown in A.

After determining the pain signal processing pattern in the frontal area, we next examined whether distinctive signals between itch and pain stimulation could be detected using NIRS topography (Fig. 8). Because a single itch experimental operation took more than 30 minutes, several types of environmental disturbances, such as sound, temperature, light, and mechanical stress, may affect the NIRS signals, we performed the experiments in a quiet room with standard control of temperature, humidity, and light intensity. After baseline measurement (Fig. 8A), the [HbO2] increased transiently in response to a scratch stimulus. An itch stimulus was applied two minutes after the scratch stimulus, after which [HbO2] increased gradually. Six minutes after the itch stimulus, the itch sensation reached maximum intensity, then gradually decreased until 10 minutes after the itch stimulus was applied. During the experiment, the skin became red when the [HbO2] peaked, then faded by 21 minutes after the itch stimulus. To determine if the itch sensation could be relieved by scratching, the scratch stimulus was applied again. The expected itch-releasing effect, however, was unclear. When the subject felt most itchy, the [HbO2] was highest. When the perception of itch had faded, the [HbO2] was relatively low but still higher than that of the resting level in all channels (Fig. 8B). VAS score of each subject showed that all subjects except for one were un-biased healthy normal subjects (Fig. S2 and S3). The agreement between perception and the [HbO2] within subjects was good. On the other hand, the change in [HbR] was somewhat larger than in the pain experiment, with a slower oscillation frequency. Except for the baseline, three other images (“Peak itch”, “Residual itch”, and “Faded itch” in Fig. 8A) exhibited a similar activation pattern, but with different intensities in the frontal area as well as in the parietal area. In contrast to pain stimulation, itch stimulation produced slower responses, which may result in a more stable imaging pattern. Based on these data, it is reasonable to deduce that the NIRS imaging pattern for gradually changing responses are fairly stable, except for the changes in intensity.

### Pattern Analysis of NIRS Imaging by Itchy Stimulation in the Prefrontal Cortex

**Figure 9:**
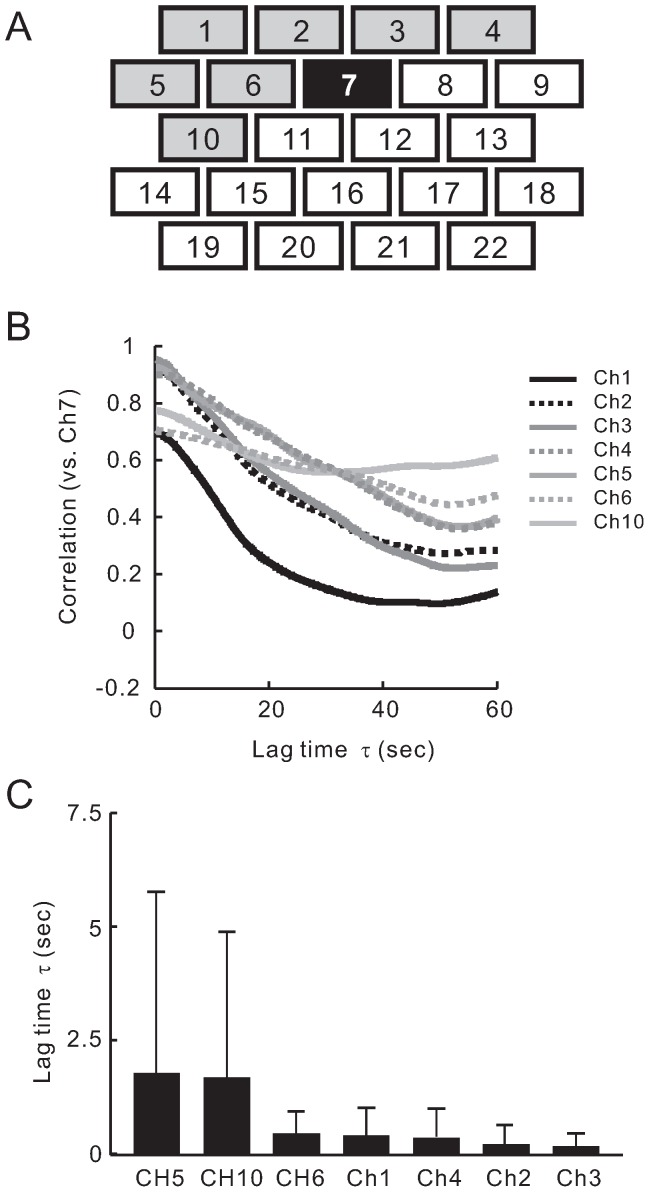
Cross correlation analysis of [HbO2] NIRS data from each channels at prefrontal area for itch stimulation. (A) Cross correlation function of [HbO2] traces from each channel against those from CH7 for pain (left) was calculated. Average correlation coefficient was 0.60, with grey channels exhibiting a significant correlation (coefficient was over 0.7). (B) Representative data shown are from subject III. No or small peak delay was observed in itch stimulation. (C) Averages of this lag time were calculated for itch stimulation (8 trials from 4 subjects). Data are shown as Mean ± SD.

As described in the pain response analysis section, cross correlation analysis was applied to [HbO2] NIRS signals for itch stimulation. The correlation between Ch7 and channels 1, 2, 3, 4, 5, 6 and 10 were significant (correlation coefficient was >0.7; averaged coefficient was 0.60; Fig. 9A). An example of cross correlation function for well correlated channels from subject III is shown in Figure 9B. No peak delay was observed in the correlated channels. Averages of delay time of each channel are summarized in Figure 9C. Results showed that [HbO2] NIRS signals changed simultaneously in these areas for itch signal processing. In other words, itch signal from the forearm might reach the prefrontal brain area and be processed at the peripheral region, in parallel to the center region, in the prefrontal cortex. The activity of prefrontal cortex for itch did not show any time-dependent connection between the central region and peripheral region of the prefrontal cortex.

### Limitations of NIRS Signals

We acknowledge that there were discrepancies in NIRS signals and the neuronal activation in vitro in which oxygenated hemoglobin and deoxygenated hemoglobin in NIRS represents an averaging in time and space over at least three orders of magnitude than what really occurs in neural networks. The NIRS is limited to about 1 second time resolution and 3 cm spatial resolution. The time scale at which a neuron is working is in the milliseconds range and the spatial extent is in the tens of micrometer range. Consequently, the NIRS techniques may have been off by approximately three orders of magnitude in both time and space. Future development of more sensitive methods might make possible a more detailed understanding of the neuronal network.

## Discussion

In the present study, we found that the activation patterns in the frontal area for pain and itch stimulation were quite distinctive and consistent within subjects across days. The peak [HbO2] in the frontal area was proportional to the intensity of perceived sensation of pain or itch, exhibiting a dose-dependent increase with increased stimulus. There was an adaptation or habituation effect of the signal intensity to repeated stimulation with the same intensity. The dynamic responses of [HbO2] and [HbT], but not [HbR] also exhibited underlying oscillations. Some areas of the frontal cortex appeared to be functionally correlated.

### Dynamic Changes in [HbO2] and [HbR]

In contrast to other types of optical imaging, [HbO2] and [HbR] signals were calculated based on a modified Beer-Lambert (absorption) law [11]. Essentially, red blood cells containing hemoglobin are flowing within the blood vessel, and capillaries, thus the velocity of HbO2 and HbR must differ due to different diameters of the inlets (the arterial end of a capillary is 5 µm) and outlets of the vascular network (the venous end of a capillary is 9 µm) [20]. If this size estimation is accurate, the velocity of HbR leaving an active area in the cortex would be about 4 times faster than that of HbO2. Assuming these arterial ends and venous ends comprise smooth muscle and can be relaxed by NO generated from the activated neurons, the blood volume ratio of the inlet to outlet may be as large or larger than 10:1. This model can explain, at least in part, why [HbO2] changes for acute pain stimulation are always delayed several tens of seconds. Of note, in all data obtained in this experiment, the change in [HbO2] was 10 times greater than that in [HbR] in the frontal area. The change in the ratio of [HbO2]/[HbR] at channels 1, 2, and 5 in the frontal area, however, was less than 3-fold in the itch experiment. The larger change in [HbR] for itch stimulation may result from stimulation with a gradually increasing intensity. This is consistent with a study showing that chronic pain induces a large change in [HbR] [21].

### Oscillations in [HbO2] and [HbR]

By smoothing the traces using the moving average, the present study found the oscillation frequency of both [HbO2] and [HbT] to be approximately 0.08 Hz. These low frequency oscillations were apparent in the frontal and parietal areas. Changes in the signals detected by NIRS topography fluctuate due to breathing (∼0.16 Hz), heart beat (∼1 Hz), and vasomotor responses (∼0.1 Hz) [22], [23]. The similarity in the frequency and phase of the [HbO2] oscillations recorded in the frontal and parietal areas suggests that the fluctuation of vasomotor activity remained in the recording. The fluctuation was not a confounding factor when the scan speed was fast.

### Coherency in the Hemodynamic Pattern in the Frontal Areas

According to the lateral views of the cortical maps proposed by Campbell [24], channels 1 through 13 (lines 1 to 3) in the array used in the present study approximately correspond to the prefrontal area and channels 14 through 22 (lines 4 and 5) correspond to the frontal area. In the parietal area, channels 1 through 10 (lines 1 to 3) correspond with the pre-central area and channels 11 through 17 (lines 4 and 5) correspond to the post-central area (intermediate), and the last line (channels 22 through 24) corresponds to a region in the parietal area. Accordingly, channels 1 through 13 in the frontal area correspond to the prefrontal cortex. The cross correlation analysis showed two types of channel connections between center of prefrontal area (Ch7) and other channels. Therefore, it is reasonable to divide the prefrontal area into two groups (Fig. 7); Ch7-correlated group (channels 1, 2, 3, 6 and 8) and Ch7-uncorrelated group. The two channel groups correspond to anatomic locations designated by Brodman area (BA) and/or Sarisov area (SA) [24]. Based on Brodman, the channels in Ch7-correlated group partially correspond to BA10, and Ch7-uncorrelated group roughly corresponds to BA9 and BA8. In the case of itch signaling, there were non-time-lag correlations between Ch7 and channels 1, 2, 3, 4, 5, 6 and 10 corresponding to BA10 (Fig. 9). Thus, our results suggest that the time-dependent correlations in BA10 are involved in pain signal processing, whereas the simultaneous activation between these areas appear to be involved in itch stimulation.

The neuro-imaging work during the last three decades have shown the SII and insular areas to be key pain integration areas [25]. In fact, a recent combined MEG-fMRI study by Mochizuki et al confirmed that itch signals are mainly located in the secondary somatosensory cortex (SII)/insula, which were also activated in the fMRI experiment by the itch stimuli[6], [26]. The somatosensory II area roughly corresponds to BA40 and 43, while insular areas roughly correspond to BA13. Our results showed that the channels in Ch7-correlated group in itch and pain experiments partially correspond to BA10.

### Different Cross Correlation Function of NIRS Signals for Pain and Itchy Stimulation

The patterns of activation following pain and itchy stimulation are similar, but distinct and quite different delay time in information processing in prefrontal cortex. It is plausible that pain and itchy are different sensation systems processed by distinct sets of neurons [26], [27], [28]. Histamine-sensitive pruriceptors have been characterized and explain the gradual histamine-induced itchy [29], [30], [31]. On the other hand, responses to acute pain lasting only a short time in our study (several tens of seconds to minutes) were not directly coupled to a physical stimulus (5 s), and subsided after cessation of the stimulus [27], [32]. Pain may be a specific sensation related to emotion, because irregularity in the responses to variable pain intensities may result from learning effects [33]. Moreover, the interaction between pain and itch is not clear. Our data showing that both scratch stimulation and pin prick stimulation evoked sharp but delayed and transient responses superimposed on a gradually increasing itchy response to histamine stimulation. Also, there was no similarity in the NIRS imaging between pain and itch. Furthermore, cross correlation analysis suggested that pain signals were processed successively, whereas itch-sensitive channels were simultaneously activated in the prefrontal region. Therefore, it can be concluded that the somatosensory signals are processed differently in the prefrontal cortex. The primary afferent neurons responsible for histamine-induced itch in humans are believed to be unmyelinated C-fibers [34]. Human C nociceptors, especially mechano-insensitive nociceptors, have a key role in generating axon-reflex erythema [35]. Schmeltz established that histamine-sensitive C fibers are included in the class of mechano-insensitive C fibers [36]. Furthermore, Apkarian discovered that the prefrontal cortex is activated by chronic pain [5]. More recently, spontaneous pain in many pain sensory-related regions has been reported to activate the prefrontal cortex [37]. The results of the present study support the prefrontal cortex activation model. Interestingly, itchy may also activate the prefrontal cortex. Changes in [HbR] were not used to create the NIRS topographic images [38]. If the dynamic change in [HbR] is also considered, a more distinct itch-sensitive pattern may be obtained.

### Conclusions

NIRS can simultaneously measure dynamic changes of [HbO2] and [HbR] in the cortex with a temporal resolution of 1 second and spatial resolution of 3 cm. The change in [HbO2] represents neural activity in the human brain. In the present study, we proposed that analyzing methods for NIRS signal could be used to determine a pain-related activation pattern in the prefrontal cortex. This was found feasible as evidenced by a highly reproducible characteristic NIRS pattern for pain and itchy stimulation among each subject. Agreement was excellent between subjective human perception and objective [HbO2] levels measured using NIRS. Non-invasive NIRS can potentially be used measure pain and itchy signal processing in awake, responsive human subjects, though large scale experiments are needed to further confirm its reliability.

## Supporting Information

Figure S1 **Dynamic frontal cortex responses and imaging after pain stimulation.** Twenty sequential NIRS images were displayed every 10 seconds. Red color indicates the region that [HbO2] NIRS signal was above baseline (increase) and blue color express the region that [HbO2] NIRS signal was under baseline (decrease).
(TIF)

Figure S2 **Direct comparison of NIRS brain images of pain and itch stimulation.** NIRS images from subjects I, II, and III when the subjects felt the most pain (A) and the most itch (B). I, II, and III corresponds to subjects F-2, F-3, and F-1, respectively.
(TIF)

Figure S3 **The distribution of VAS scores.**
(TIF)

Text S1 **NIRS responses in frontal area after pain stimulation.**
(DOC)

Text S2 **Distinctive topographic imaging of pain compared with that of itch.**
(DOC)
