# Supplementary figures and images for: Analysis for Distinctive Activation Patterns of Pain and Itchy in the Human Brain Cortex Measured Using Near Infrared Spectroscopy (NIRS)
Source: PLoS One. 2013 Oct 3;8(10):e75360. doi: 10.1371/journal.pone.0075360 (PMC3789686; doi:10.1371/journal.pone.0075360)

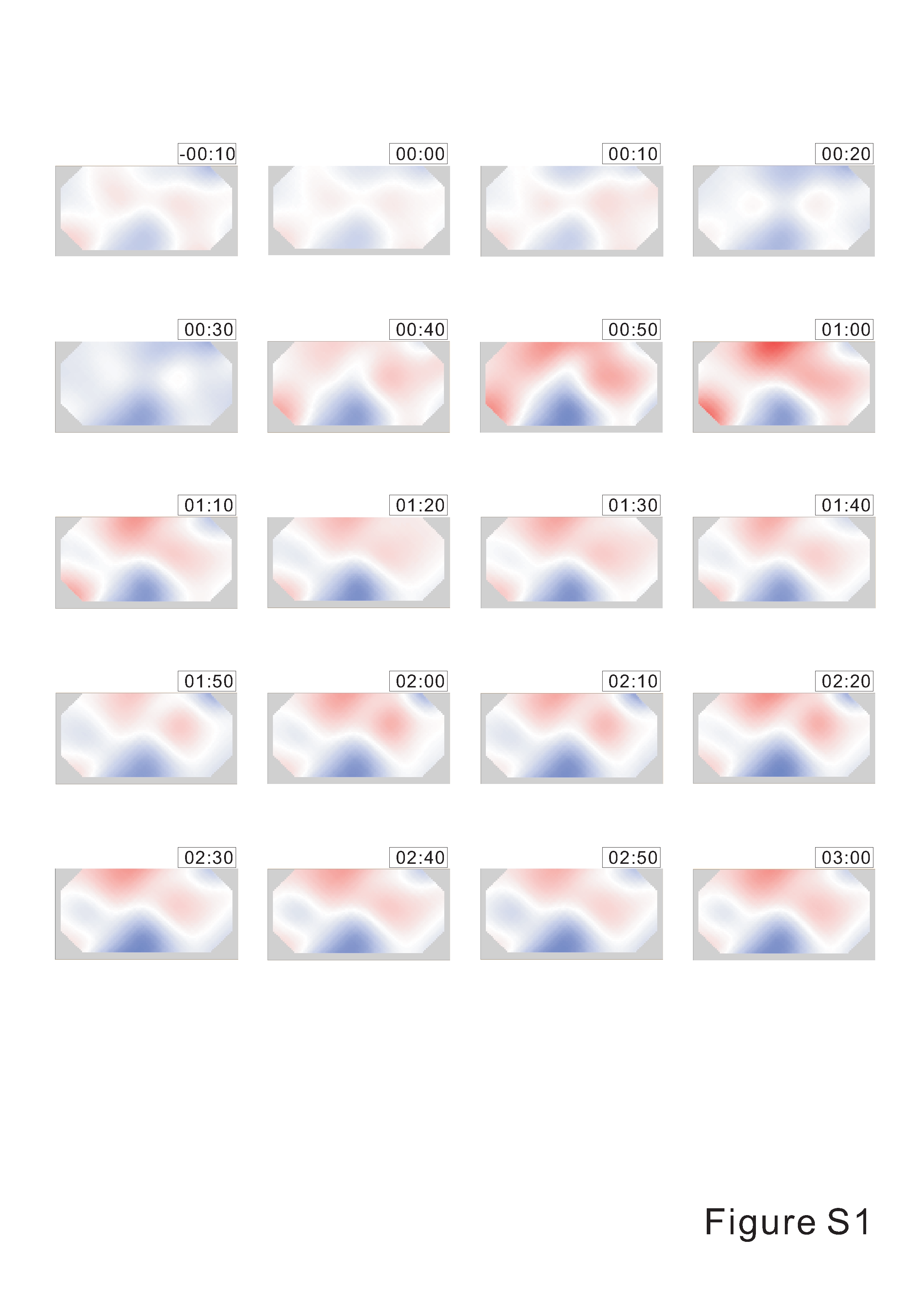

Supplement: Figure S1 — Dynamic frontal cortex responses and imaging after pain stimulation. Twenty sequential NIRS images were displayed every 10 seconds. Red color indicates the region that [HbO2] NIRS signal was above baseline (increase) and blue color express the region that [HbO2] NIRS signal was under baseline (decrease). (TIF) [file pone.0075360.s001.tif]

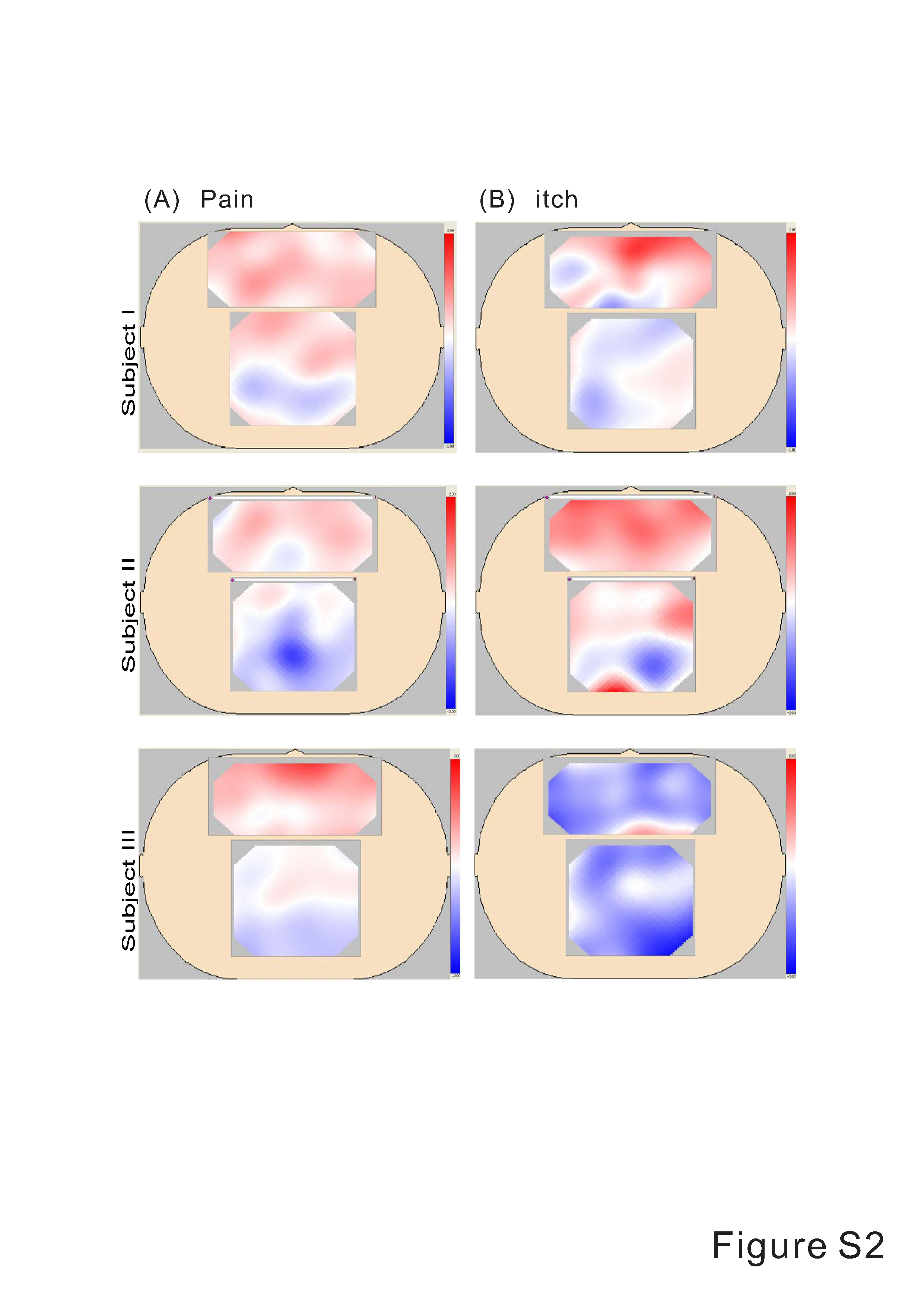

Supplement: Figure S2 — Direct comparison of NIRS brain images of pain and itch stimulation. NIRS images from subjects I, II, and III when the subjects felt the most pain (A) and the most itch (B). I, II, and III corresponds to subjects F-2, F-3, and F-1, respectively. (TIF) [file pone.0075360.s002.tif]

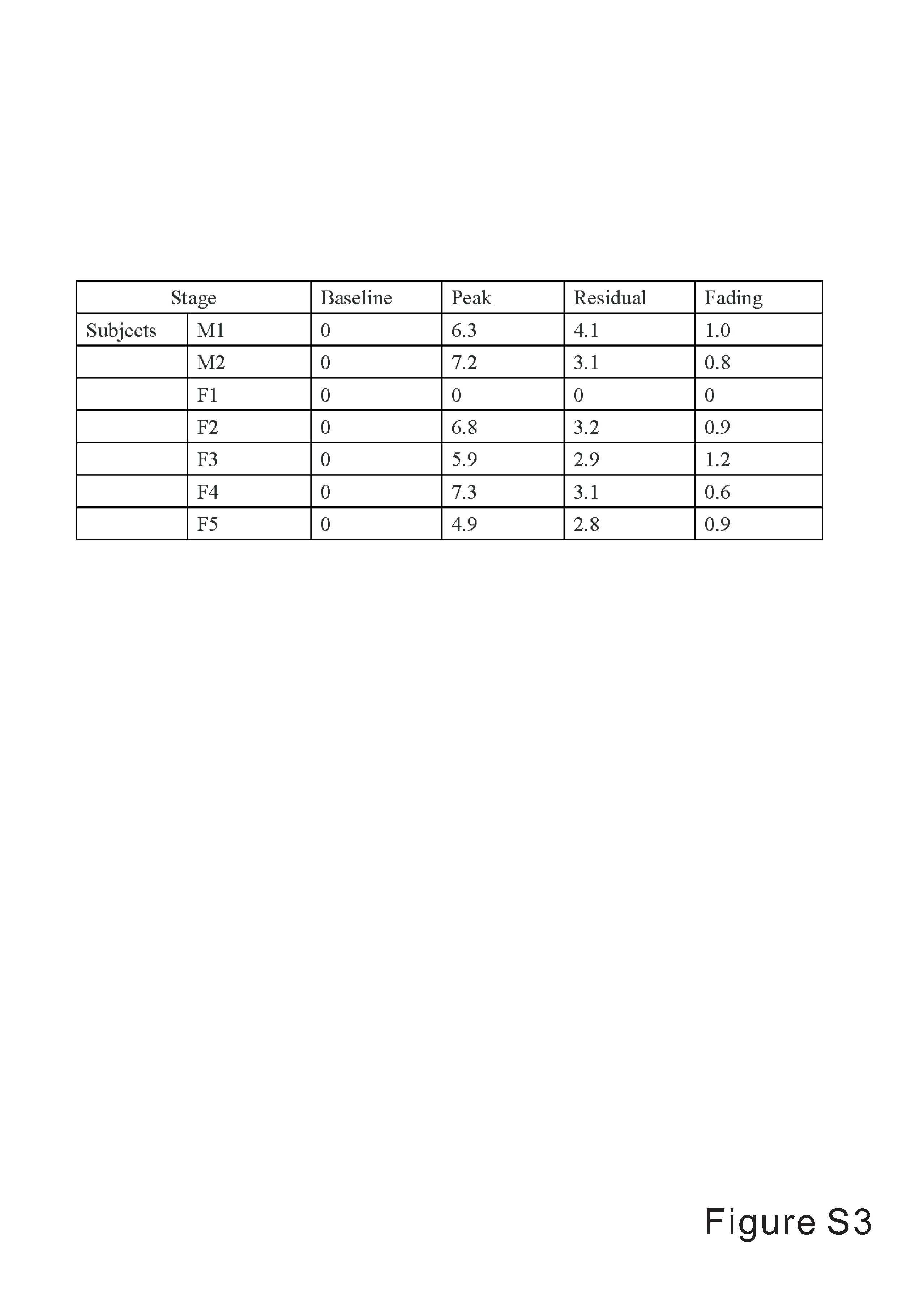

Supplement: Figure S3 — The distribution of VAS scores. (TIF) [file pone.0075360.s003.tif]
