# Supplementary material for: Analysis for Distinctive Activation Patterns of Pain and Itchy in the Human Brain Cortex Measured Using Near Infrared Spectroscopy (NIRS)
Source: PLoS One. 2013 Oct 3;8(10):e75360. doi: 10.1371/journal.pone.0075360 (PMC3789686; doi:10.1371/journal.pone.0075360)
Supplement: Text S1 — NIRS responses in frontal area after pain stimulation. (DOC) [file pone.0075360.s004.doc]

NIRS responses in frontal area after pain stimulation

Twenty serial images for subject (IV) based on the optical data from channels of the frontal area were represented every 10 sec before and after pain stimulation (Fig. S1). NIRS signals changed dynamically after pain stimulation in the frontal area. Results were confirmed by PET imaging (data not shown).
