# Supplementary material for: Analysis for Distinctive Activation Patterns of Pain and Itchy in the Human Brain Cortex Measured Using Near Infrared Spectroscopy (NIRS)
Source: PLoS One. 2013 Oct 3;8(10):e75360. doi: 10.1371/journal.pone.0075360 (PMC3789686; doi:10.1371/journal.pone.0075360)
Supplement: Text S2 — Distinctive topographic imaging of pain compared with that of itch. (DOC) [file pone.0075360.s005.doc]

Distinctive topographic imaging of pain compared with that of itch

Direct comparison of NIRS topography demonstrates a different activation pattern for pain and itch stimulation within subjects (Fig. S2). Each individual subject exhibited a specific pattern of activity, not only in the frontal, but also in the parietal area. Among them, subject F-1 was the most interesting because this subject appeared to process pain sensation but not itch. The actual perception of this subject based on the VAS scale was consistent with these results (Fig. S3). These results suggested that NIRS topographic imaging reflects the actual subjective pain or itch sensation.
